# An Upscaling Method for Cover-Management Factor and Its Application in the Loess Plateau of China

**DOI:** 10.3390/ijerph10104752

**Published:** 2013-10-09

**Authors:** Wenwu Zhao, Bojie Fu, Yang Qiu

**Affiliations:** 1 State Key Laboratory of Earth Surface Processes and Resource Ecology, College of Resources Science and Technology, Beijing Normal University, 19 Xinjiekou Outer Street, Beijing 100875, China; 2 State Key Laboratory of Urban and Regional Ecology, Research Center for Eco-Environmental Sciences, Chinese Academy of Sciences, 18 Shuangqing Road, Beijing 100085, China; E-Mail: bfu@rcees.ac.cn; 3 School of Geography, Beijing Normal University, 19 Xinjiekou Outer Street, Beijing 100875, China; E-Mail: qiuyang69@bnu.edu.cn

**Keywords:** cover-management factor, scale, soil erosion, rainfall erosivity, Universal Soil Loss Equation (USLE), soil conservation, C-factors

## Abstract

The cover-management factor (C-factor) is important for studying soil erosion. In addition, it is important to use sampling plot data to estimate the regional C-factor when assessing erosion and soil conservation. Here, the loess hill and gully region in Ansai County, China, was studied to determine a method for computing the C-factor. This C-factor is used in the Universal Soil Loss Equation (USLE) at a regional scale. After upscaling the slope-scale computational equation, the C-factor for Ansai County was calculated by using the soil loss ratio, precipitation and land use/cover type. The multi-year mean C-factor for Ansai County was 0.36. The C-factor values were greater in the eastern region of the county than in the western region. In addition, the lowest C-factor values were found in the southern region of the county near its southern border. These spatial differences were consistent with the spatial distribution of the soil loess ratios across areas with different land uses. Additional research is needed to determine the effects of seasonal vegetation growth changes on the C-factor, and the C-factor upscaling uncertainties at a regional scale.

## 1. Introduction

The Universal Soil Loss Equation (USLE) enables planners to predict the average rate of soil erosion for each feasible alternative crop system and management combination in association with specific soil types, rainfall patterns, and topography [1]. The soil loss equation is A = R × K × L × S × C × P, where A is the computed soil loss per unit area, R is the rainfall erosivity factor (R-factor), K is the soil erodibility factor (K-factor), L is the slope-length factor (L-factor), S is the slope-steepness factor (S-factor), C is the cover and management factor (C-factor), and P is the support practice factor (P-factor). Among the USLE factors, the C-factor is perhaps the most important because it represents conditions that can be easily managed to reduce soil erosion. The C-factor is the ratio of the soil loss from land with a specific type of land use/land cover to the soil loss from continuously fallow and tilled land under the same environmental conditions. The C-factor is unitless, with a value between 0 and 1. Larger C-factor values indicate that the corresponding land cover type results in more soil erosion than the other land cover types, which reflects the comprehensive effects of the C-factor on soil erosion [1,2,3,4]. Systematic C-factor studies began in the United States in the 1950s, when a large numbers of models and model parameters were established and validated [1,2,3,5,6]. Work by Wischmeier [7] and Mutchler *et al.* [8] indicated that the general influences of cropping and management systems on soil loss could be divided into a series of subfactors. Within the Revised Universal Soil Loss Equation (RUSLE), the soil loss ratio (SLR) values were calculated for each period before weighting by the R-factor fraction for each period. These weighted values were combined into an overall C-factor value [3]. The USLE model was introduced to China in the 1980s [9]. Since the 1980s, C-factor studies have been conducted in China at a plot scale, and SLRs have been measured for different crop stages [10,11,12,13]. Watershed- or regional-scale estimations of the C-factor have primarily been based on plot-scale experimental data for different land use or vegetation types [10,11,12,14,15]. However, at a large scale, different crop types cannot be identified (in most cases) due to the low resolution of the land use/cover maps. In addition, some crop’s C-factor values were often used to represent the C-factor of farmland at a watershed- or regional-scale. However, small-scale study results may not accurately or directly apply to large-scale studies [16,17], which can affect the accuracy of C-factor estimation at large scales [18]. Thus, it is important to determine methods for calculating the C-factor values at a large scale. These methods will provide an important basis for large-scale soil erosion evaluations. Scaling refers to the process of making inferences about a variables behavior at a given scale based on information and knowledge at another scale [19,20]. Scaling is classified as either upscaling or downscaling based on the direction of the conversion. Upscaling refers to the extrapolation of information from a small scale to a large scale in the process of information aggregation. Downscaling refers to the extrapolation of information from a large scale to a small scale in the process of information decomposition [21,22]. In the scaling process, different research designs are required for different scenarios [23]. For example, when a similar mechanism is applicable for multiple models that are operating at different scales, the conversion between scales is achieved by modifying the grain and/or extent of the model [24].

The hilly area in the loess plateau region of China potentially has the highest erosion rate in the world [25]. This region is mainly located in the northern areas of the Shanxi and Shaanxi Provinces. Here, we used one county (Ansai County) in northern Shaanxi to test the use of upscaling for obtaining a regional C-factor. We used the scaling concept based on the slope-scale computational equation to derive an upscaling method for estimating the C-factor at a regional scale. This method provides useful information that supports the assessment of large-scale soil erosion in the loess plateau of China and provides a new application of upscaling in soil erosion studies. 

## 2. Materials and Methods

### 2.1. Study Area

Ansai County in Shaanxi Province, China, was chosen as the study area. Ansai County is located in the central region of the loess plateau in northern Shaanxi at 108°51'44''–109°26'18'' longitude, 36°30'45''–37°19'03'' latitude. This region covers approximately 2,950 km^2^ and has an average altitude of 1,371.9 m and an annual average temperature of 8.8 °C. The climate in this region is classified as temperate continental semi-arid monsoon with an annual average precipitation of 505.3 mm. This precipitation is characterized by significant interannual variations and is unevenly distributed throughout the year. The precipitation in July, August and September accounts for approximately 63% of the annual precipitation. In addition, this region has uneven topography that is typical of China’s loess hill and gully region. Specifically, this region consists of intertwined gullies with a gully density of 4.33 km/km^2^. The region has low vegetation cover primarily composed of *Artemisia sp.* and *Pennisetum sp. (*ground species), *Rosaceae* *sp.* and *Lespedeza sp*. (shrub species). Generally, trees are found in natural and planted secondary forests [26]. There are 12 townships in Ansai County, including Gaoqiao Xiang, Wangjiawan Zhen, Yanhewan Zhen, Louping Xiang, Wangyao Xiang, Zhenwudong Zhen, Liandaowan Xiang, Zhao’an Zhen, Pingqiao Zhen, Huaziping Zhen, Jianhua Zhen, and Zhuanyaowan Zhen. These towns cover areas of 128.09, 186.21, 208.31, 218.67, 224.35, 226.84, 228.03, 264.46, 266.10, 301.59, 305.42, and 392.31 km^2^, respectively.

### 2.2. Study Methods

The calculation of the C-factor value at a large scale should be based on small-scale research results. On one hand, the C-factor calculated at a regional scale should be consistent with the C-factor calculated at a plot scale. On the other hand, the SLR values of different crops at the plot scale and regional rainfall data (from local rain gauges) can provide the data that are necessary for calculating the C-factor at a regional scale.

#### 2.2.1. Basis for the C-Factor Value Calculations at a Regional Scale

At the slope scale, the C-factor value can be calculated based on the seasonal rainfall erosivity distribution and the SLR at different growth stages as follows [3,27]:

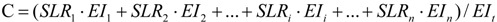
(1)
where C is the cover-management factor at the plot scale, *SLR*_i_ is the soil loss ratio for the time period *i*, *EI*_i_ is the percentage of EI during the time period *i*, n is the number of periods used in the summation, and *EI*_t_ is the sum of the EI percentages for the entire study period. In addition, EI is the kinetic energy of the rainfall (E) multiplied by the rainfall intensity (I). The maximum 30 min rainfall intensity (I_30_) is often used to calculate EI. 

Based on the plot-scale C-factor calculation equation, the estimation of the C-factor value at a regional scale should consider the rainfall erosivity and SLR. While, the spatial variability of each rainfall event is very large in the loess plateau region of China, and monthly rainfall erosivity calculations are more suitable than individual rainfall erosivity calculations at a regional scale. A period of one month was used for rainfall erosivity calculation. Consequently, the soil loss ratio should be calculated on a monthly basis. Thus, based on the slope scale equation, the regional C-factor can be calculated by using a monthly soil loss ratio for the different land cover types and monthly rainfall erosivity values. However, unlike the slope-scale calculation equation, the monthly data for the soil loss ratio and rainfall erosivity equations are not “point data”. Rather, these data are spatial data that reflect spatial variations of the soil loss ratios and rainfall erosivity across the study area.

#### 2.2.2. The C-factor Estimation Equation at a Regional Scale

Based on the above analysis, the calculation equation for the regional C-factor values was described as follows:

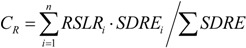
(2)
where *C_R_* is the cover-management factor at a regional scale, *RSLR_i_* is the soil loss ratio at a regional scale for month *i*, *SDRE_i_* is the rainfall erosivity at a regional scale for month *i*, n is the number of months used in the summation, and Σ*SDRE* is the sum of the regional rainfall erosivity values across the entire study period.

##### Estimation of Rainfall Erosivity at a Regional Scale

Rainfall erosivity was calculated as the product of E and I. However, because E and I vary across a region, large amounts of rainfall data were needed to calculate rainfall erosivity as a product of E and I. Here, the relationship between monthly rainfall erosivity and monthly precipitation was used to calculate the rainfall erosivity for each rain gaug station. The calculation equation was derived from the rainfall data of 10 rain gauges in the Yanhe watershed (located in northern Shaanxi). Based on the individual rainfall data of the ten rain gauges between 1981 and 1989, the EI_30_ values were calculated based on the RUSLE handbook instructions. In addition, the monthly EI_30_ values were calculated as the sum of the EI_30_ values of each erosive storm that occurred during the month. In addition, the Rain_9_ (the monthly rainfall for days with ≥9.0 mm) values were derived from the rain gauge data. Different models were tested, and the regression equation for the R-factor and rain_9_ was derived as follows (R^2^ = 0.626) [28]:

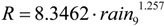
(3)
where R is the monthly rainfall erosivity (MJ·mm/km^2^·h) and *rain*_9_ represent the monthly sum of the daily precipitation (mm) on the days with a daily precipitation of more than 9 mm.

The rainfall erosivity can be estimated for each rainfall gauging station within the study area using Equation (3). In addition, the spatial distribution of the rainfall erosivity (*SDRE*) at a regional scale can be determined from the spatial interpolation of the rainfall erosivity by using a geographic information system (GIS). The spatial distribution of the annual rainfall erosivity (Σ*SDRE*) was calculated by summing the spatial distribution maps of the rainfall erosivity data from different months.

##### Monthly Soil Loss Ratio Estimation at a Regional Scale

The monthly soil loss ratios were obtained for different land cover types at a regional scale, which including the soil loss ratio for farmland (*SLR_F*) and the soil loss ratio for other land cover types (*SLR_O*).

(1) Soil loss ratios for farmland at a regional scale

At the plot scale, previous studies in China have estimated the soil loss ratios for different crops [11,13]. At a regional scale, the SLR value map for farmland may be directly derived from a crop type map. However, due to the low resolution of the land use map at a regional scale, the different crop types could not be identified. To estimate the soil loss ratios for farmland at a regional scale, the percentages of crop types were used to weight the C-factors of the different crop types. The regional soil loss ratios for farmland can be calculated by using the following equation:

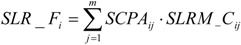
(4)
where *SLR_F_i_* is the soil loss ratio for farmland during month *i*, *SLRM_C_ij_* is the soil loss ratio for crop *j* during month *i*, *SCPA_ij_* is the percentage of the planting area of crop *j* during month *i* and m is the number of crop types.

(2) Soil loss ratios for the other land cover types at a regional scale

Few studies have reported the soil loss ratios for different growth stages of woodlands, grasslands or other land cover types at a plot scale in China. Thus, unlike the soil loss ratios for farmland, the soil loss ratios for other types of land cover, including woodlands and grasslands, were not calculated from the monthly data. Instead, annual average data from relevant literature were used to calculate these soil loss ratios [10,11,12,15]. The monthly soil loss ratios for farmland (*SLR_F*) and the soil loss ratios for other land cover types (*SLR_O*), such as woodlands and grasslands, were imported into the land use database to obtain the regional-scale spatial distribution of the soil loss ratios for different monthly land use/cover types by month, expressed as the soil loss ratio for month *i* at the regional scale (*RSLR_i_*).

#### 2.2.3. Data Sources

##### Precipitation Data

**Figure 1:**
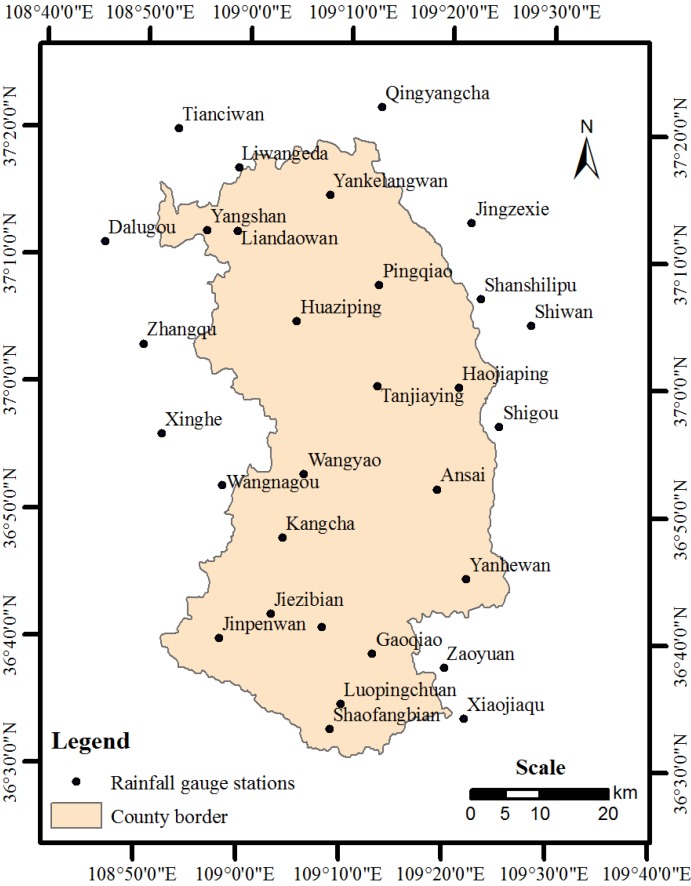
Locations of the rainfall gauging stations in the study area.

Rainfall erosivity was calculated for each rainfall gauging station using Equation (3) with the daily precipitation data from the rainfall gauging stations. The daily precipitation data were from collected between 1981 and 1989 and were recorded in the “Hydrological Yearbook of People’s Republic of China—Hydrological Data of Yellow River Basin” [29]. The selected rainfall gauging stations are shown in Figure 1.

##### Soil Loss Ratio Data

The monthly soil loss ratio for the crops in the loess area and the percentage of planted area for each crop in the different townships of Ansai County were used to calculate the soil loss ratios of the farmland. The percentage of planted area according to crop type was determined based on the planted areas of the different crops in the Ansai County townships using data from the *Basic Economic Statistical Data of Ansai County (1981–1989)* [30]. The monthly soil loss ratio data for each crop type were obtained from published research findings [11,13]. These research studies were conducted in Ansai (Shanxi Province) and Tianshui counties (Gansu Province). The main crop types included soybean, potato, buckwheat, maize, winter wheat, and others. The 156 plot year observation data were used to calculate the soil loss ratio from the different types of crops and fallow land. The crop year was divided into the following six periods based on the change in crop coverage with time: fallow (from plowing to preparing the seedbed), seedbed (preparing the seedbed to obtaining 10% coverage), established (10% to 50% coverage), development (50% to 75% coverage), mature (75% to harvest), and residue and stubble (harvest to plowing). Because the soil loss ratios for crops were previously measured for the crop-growing season, the monthly weighted soil loss ratio was used in this study. The soil loss ratio data for other types of land cover, such as woodland and grassland, were obtained from the literatures [10,13,14]. Because plot experiments were lacking, the SLR values of the woodlands and grasslands were directly based on plot research results and vegetation coverage. 

##### Land Use/Land Cover Data

The ERDAS IMAGINE 8.5 software was used for the geometric correction and projection transformations of the Landsat TM remote sensing data that were collected from the study area. Spectrum enhancement was performed by changing the principal components to reduce data redundancy and eliminate noise. A human-computer interactive mode was used to interpret the land use/cover data. The land use/cover types included farmland, forestland, shrubland, woodland, other timberland, high-cover grassland, moderate-cover grassland, low-cover grassland, water bodies, and residential and built-up lands.

## 3. Results

### 3.1. Rainfall Erosivity at a Regional Scale

**Figure 2:**
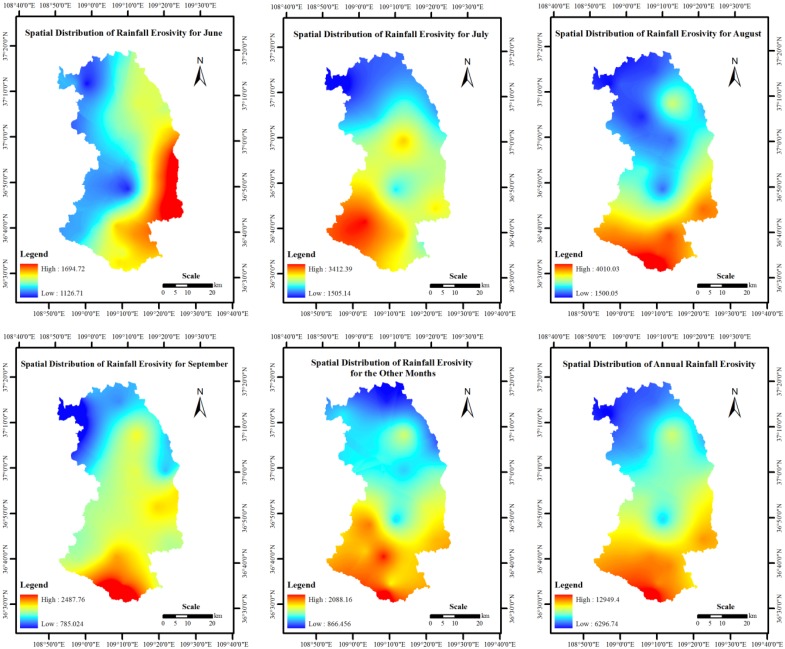
Spatial distributions of monthly and annual rainfall erosivity in Ansai County.

Based on the annual distribution of rainfall in Ansai County, each year was divided into 5 study periods, including June, July, August, September and other months outside the rainy season. The multi-year mean monthly rainfall erosivity and mean annual rainfall erosivity were calculated for each rainfall gauging station using Equation (3). The daily precipitation data were collected from the rainfall gauging stations in the study area. Four multivariate geostatistical methods were used to interpolate the rainfall erosivity values. By comparing prediction errors from different methods, it was observed that the mapped surfaces from disjunctive kriging provided more accurate rainfall erosivity predictions than ordinary kriging, universal kriging and simple kriging (Table 1). However, the prediction errors were not perfect. The disjunctive Kriging method was used to interpolate the rainfall erosivity spatially to obtain the spatial distribution of the multi-year mean monthly rainfall erosivity and the mean annual rainfall erosivity for the study period (Figure 2). As shown in Figure 2, the rainfall erosivity in Ansai County was greater in July and August. The annual rainfall erosivity distribution in the study area indicated that the erosivity was lower in the north and higher in the south.

**Table 1:** Prediction errors of average annual rainfall erosivity from the different kriging methods.

Algorithms	Prediction errors			
	Mean	Root-mean-square	Average standardized error	Root-mean-square standardized
Ordinary	−8.742	870.4	1020	0.8824
Simple	8.635	903	1109	0.8382
Universal	2.376	207.1	264.3	0.8131
Disjunctive	−1.468	203.4	203.2	1.205

### 3.2. Soil Loss Ratios

#### 3.2.1. Soil Loss Ratios for Farmland

The weighted SLR values for different crops in June, July, August, September and the other months were based on the previously reported SLR values for different growth stages (Table 2). The farmland SLR values for each township in Ansai County were calculated using Equation (4). These results are shown by township in Table 3.

No significant differences were observed between the farmland soil loss ratios of the townships (Table 3). However, clear monthly differences were observed. The SLR values were relatively low in August and September and increased in the months outside of the rainy season. In addition, the changing SLR values were strongly correlated with the crop growth cycle. From July to September, the crop growth was rapidly, which resulted in high vegetation coverage and protected the soil from the direct impact of rainfall. The farmland soil loss ratios under these vegetation conditions were significantly lower than the ratios for continuously fallow land. This difference resulted in relatively low farmland soil loss ratios from July to September. In contrast, the vegetation cover during the other months was relatively low and the farmland soil loss ratios were not significantly lower than the soil loss ratios of the continuously fallow land. Therefore, the SLR values during the other months were significantly greater than the SLR values from July to September.

**Table 2:** Monthly soil loss ratio by crop type.

Crop type	June	July	August	September	Other months
Wheat	0.17	0.19	0.21	0.50	0.23
Summer potato	0.84	0.51	0.40	0.30	0.75
Maize	0.45	0.40	0.39	0.41	0.59
Sorghum	0.52	0.49	0.45	0.43	0.72
Millet	0.54	0.52	0.52	0.52	0.93
Proso millet	0.58	0.52	0.50	0.54	0.76
Autumn tuberous crop	0.81	0.50	0.39	0.31	0.74
Buckwheat	1.00	0.89	0.64	0.22	0.61
Other miscellaneous grains	0.83	0.64	0.52	0.43	0.62
Soybean	0.68	0.54	0.46	0.46	0.64
Economic crops	0.49	0.53	0.54	0.57	0.78
Other crops	0.51	0.45	0.44	0.47	0.66

**Table 3:** Monthly farmland soil loss ratios in the townships of Ansai County.

Township	Farmland soil loss ratio				
	June	July	August	September	Other months
Zhuanyaowan Zhen	0.58	0.51	0.46	0.45	0.66
Gaoqiao Xiang	0.55	0.49	0.45	0.46	0.65
Louping Xiang	0.55	0.48	0.44	0.46	0.65
Yanhewan Zhen	0.56	0.49	0.44	0.46	0.66
Zhaoan Zhen	0.55	0.48	0.44	0.46	0.65
Wangyao Xiang	0.55	0.49	0.45	0.46	0.66
Zhenwudong Zhen	0.54	0.47	0.45	0.47	0.68
Jianhua Zhen	0.54	0.47	0.43	0.46	0.62
Huaziping Zhen	0.55	0.48	0.44	0.46	0.64
Liandaowan Xiang	0.58	0.50	0.46	0.46	0.66
Pingqiao Zhen	0.55	0.48	0.44	0.46	0.64
Wangjiawang Xiang	0.56	0.49	0.44	0.46	0.65

#### 3.2.2. Soil Loss Ratios for the Other Types of Land Cover

Due to the lack of plot scale experimental data for computing the soil loss ratios for woodlands, grasslands, residential lands and lands with other types of land use/ cover in the loess plateau of China, the annual average SLR values for lands with other cover types were obtained from previous studies (Table 4). Based on these previous studies, the low-cover grasslands and other timberland areas generally have greater SLR values, while forestlands and high-cover grasslands have lower SLR values (Table 4). These SLR values were closely related to differences in vegetation coverage.

**Table 4:** Annual average soil loss ratio according to type of vegetation coverage.

Land cover type	Soil loss ratio
Forest	0.09
Shrubland	0.22
Woodland	0.15
Other timberland	0.31
High-cover grassland	0.12
Moderate-cover grassland	0.18
Low-cover grassland	0.32
Water body	0.00
Residential and built-up land	0.20

#### 3.2.3. Soil Loss Ratios for Ansai County

**Figure 3:**
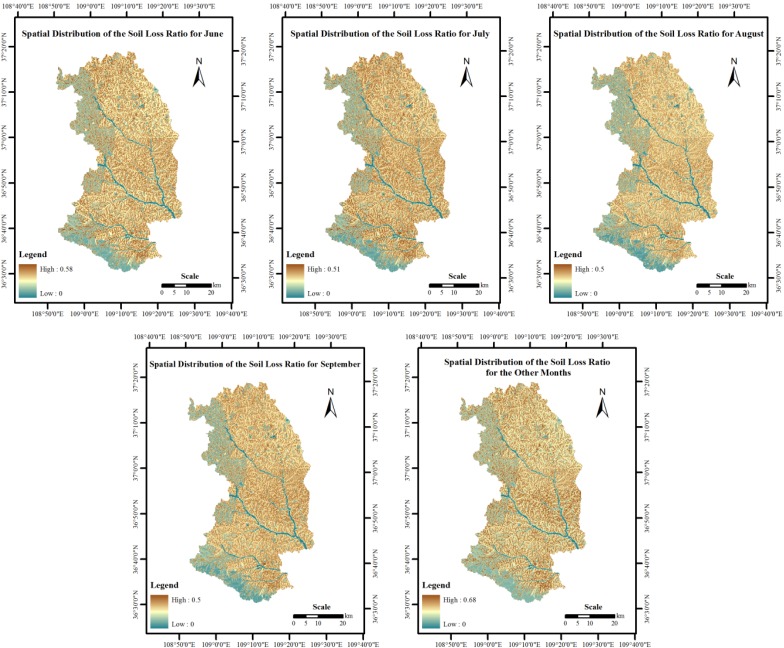
Spatial distribution of the monthly soil loss ratios in Ansai County.

A spatial analysis module (in the ArcGIS software) was used to obtain the soil loss ratio by land cover type in Ansai County and to plot the distribution of the SLR values for the different study periods. This ratio was based on the soil loss ratio data in Table 3 and Table 4 and on the land use/cover map for the study period (Figure 3). As shown in Figure 3, clear temporal and spatial differences occurred between the soil loss ratios of the areas with different types of land coverage. The annual soil loss ratio for the eastern region was greater than the annual soil loss ratios for the western and southern regions. In addition, the monthly soil loss ratios in July, August and September were lower than that in the other months.

### 3.3. The Cover-Management Factor at the County Scale

**Figure 4:**
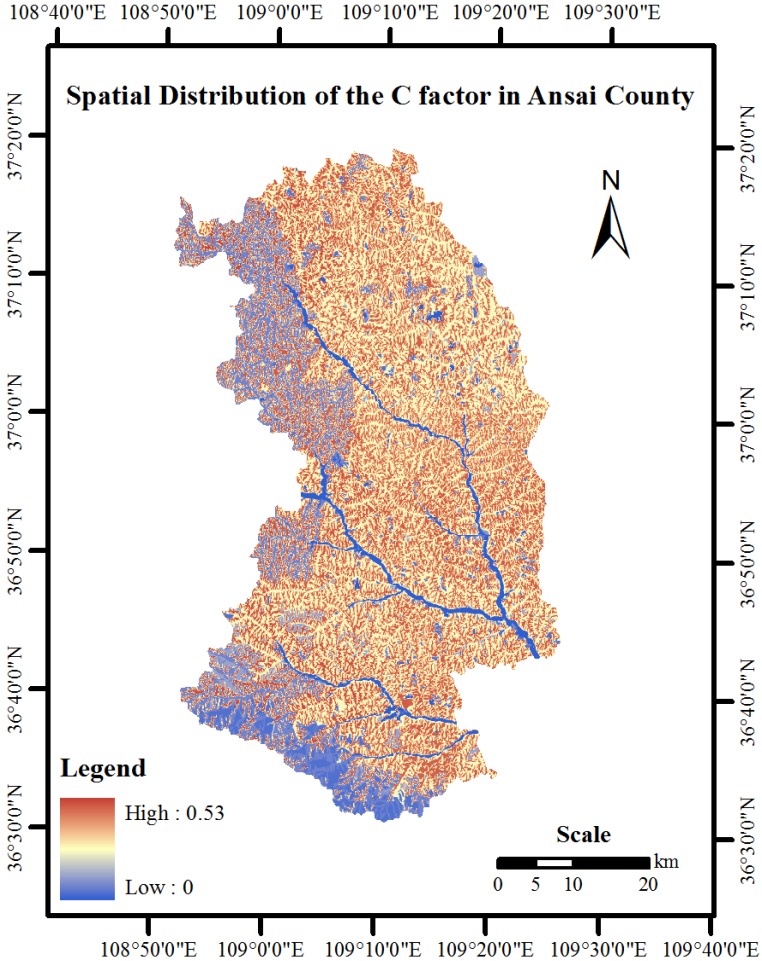
Spatial distribution of the cover-management factor in Ansai County.

A spatial distribution map of the C-factors for Ansai County was developed for the 1980s by applying Equation (2) and the ArcGIS spatial analysis module (Figure 4). The multi-year average monthly rainfall erosivity data, the annual rainfall erosivity data (Figure 2) and the spatial distribution of the monthly soil loss ratio were used (Figure 3). The average and maximum C-factors for Ansai County were 0.36 and 0.56, respectively (Figure 4). Regarding the spatial distribution, the C-factor for the eastern region was slightly greater than the C-factor for the western region. This spatial difference was consistent with the spatial distribution of the soil loss ratio with land use type. However, because the C-factor is affected by the spatial and temporal distribution of rainfall erosivity, there were no significant differences for the C-factor from east to west. In addition, because woodlands accounted for most of the land cover in the southern portion of Ansai County, the C-factor in this region was significantly lower relative to the other regions.

## 4. Discussion and Conclusions

Here, we used Ansai County as a study area to determine a method for calculating the C-factor at a regional scale. To accomplish this task, we modified the C-factor calculation equation at the slope scale. In addition, the C-factor was calculated for Ansai County by combining existing experimental plot data for soil loss ratios with regional rainfall data. Overall, following results were obtained: (1) after altering the equation parameters, it was possible to estimate the C-factor on a regional scale based on the equation for the plot scale, (2) the farmland soil loss ratios in Ansai County were relatively low in August and September, and significantly greater in the months outside of the rainy season, and (3) the average and maximum C-factors for the county were 0.36 and 0.56, respectively, the C-factor values were greater in the eastern region than in the western region and the C-factor values were the lower along the southern border.

Relative to previous studies that calculated C-factor in China (assigning the farmland’s C-factor value at a regional scale to the crop’s C-factor value at a plot scale), the regional-scale C-factor values for farmland that were obtained by calculation in this study better reflected the different SLR values and percentages of the different crops. In addition, the seasonal rainfall erosivity and SLR values that were estimated for different land use types were considered. These values can potentially improve the accuracy of the regional C-factor. However, multiple uncertainties were introduced in this study. First, the rainfall erosivity maps were derived by the disjunctive kriging method after estimating the rainfall erosivity values with the regression equation (Equation (3)). The cumulative errors of this equation and the interpolation method may affect the regional C-factor values. In addition, the estimated regional soil loss ratios do not consider the seasonal variations of the vegetation growth because soil loss ratio data for woodlands and grasslands, and other crop types are limited. Thus, the accuracy of regional C-factor values is limited. Finally, vegetation coverage can affect the SLR value, but this effect was not addressed in this study.

The regional-scale C-factor plays an important role in the predicting and assessing large-scale soil erosion. The impacts of seasonal vegetation growth on the C-factor should be estimated in the future studies. Thus, experimental monitoring and the application of multi-source remote sensing data for estimating and analyzing regional C-factor values must be improved. For a given type of land use, the soil loss ratios will differ if the soil type, topography, or soil and water conservation practices change. Therefore, additional plot experiments should be conducted and additional attention should be given to how the K-, S-, L- and P- factors can be used to determine the C-factor and soil loss ratio. Finally, scaling modularization with GIS could potentially facilitate the application and popularization of the scaling method.
